# The Effects of the COVID-19 Pandemic on Mental Health Among Older Adults From Different Communities in Chengmai County, China: Cross-sectional Study

**DOI:** 10.2196/37046

**Published:** 2022-05-06

**Authors:** Zhimin Xu, Gabriela Lima de Melo Ghisi, Lixian Cui, Fang Zeng, Xiaohai Zhou, Zhongtang Yue, Hanbei Chen

**Affiliations:** 1 Department of Cardiology Xinhua Hospital Affiliated to Shanghai Jiao Tong University School of Medicine Shanghai China; 2 University Health Network Toronto, ON Canada; 3 Division of Arts and Sciences New York University Shanghai Shanghai China; 4 Department of Nursing People’s Hospital of Chengmai County Jinjiang Town China; 5 Department of Endocrinology Xinhua Hospital Affiliated to Shanghai Jiao Tong University School of Medicine Shanghai China; 6 Coconut Town Community College of Elderly Jinjiang Town China

**Keywords:** mental health, COVID-19, depression, anxiety, aged, aging, older adults

## Abstract

**Background:**

Due to the strict measures employed to control the spread of SARS-CoV-2, the extent of COVID-19 goes beyond morbidity and mortality and affects individuals’ mental health in the long term.

**Objective:**

This cross-sectional study aimed to investigate the effects of the COVID-19 pandemic on mental health and its contributing factors among older people in Chengmai County, China.

**Methods:**

A web-based survey was administered through WeChat between March and April 2020. Older people (ie, >50 years) from local and foreign community groups completed the survey, which included items on sociodemographic and clinical characteristics, the 7-item Generalized Anxiety Disorder scale (GAD-7), and the 9-item Patient Health Questionnaire (PHQ-9). Independent *t* tests and a multiple linear regression analysis were used to investigate differences between anxiety and depression and the factors associated with these symptoms across the 2 groups.

**Results:**

Overall, 469 responses were received; 119 responses (25.4%) were from male participants and 202 (43.1%) were from those older than 65 years. Of the 469 responses, 245 (52.2%) were from the local community group and 224 (47.8%) from the foreign group. The mean GAD-7 (*P*=.003) scores were significantly higher in the local group. Anxiety was significantly more present in the local group (61/245, 24.9% compared to 35/224, 15.6% in the foreign group; *P*=.01). A total of 6 respondents presented severe anxiety and 2 presented severe depression.

**Conclusions:**

This study demonstrated that both community groups of older adults from the Chinese “Hometown of Longevity” presented anxiety or depressive disorders during the first months of the pandemic. Local community groups presented significantly more mental health disorders, which were associated with a history of previous psychological disorders.

## Introduction

SARS-CoV-2 is a novel coronavirus identified as the cause of COVID-19 that emerged in Wuhan, China, in late 2019 and spread worldwide [1]. As of January 17, 2022, approximately 330 million cases have been confirmed worldwide, with 5.5 million deaths [2]. Due to its highly contagious pathogenic nature, safety measures employed by governments worldwide have tried to prevent the spread of COVID-19; these measures include social distancing (ie, limiting social gatherings), avoiding in-person interactions, and lockdowns [3]. Due to these strict measures, the effects of COVID-19 go beyond morbidity and mortality, and the long-term impact on mental health has been heavily discussed in clinical, scientific, and political settings [4-7].

Although COVID-19 can affect persons of any age, older people are particularly vulnerable to serious infection and death [8,9]. This group has been advised to stay indoors, avoid contact with family and friends, limit in-person visits, and have groceries and medicines delivered to their homes or shop during specific hours to reduce their risk of infection [10,11]. Before the pandemic, poor mental health in the elderly community was already considered a serious problem, with approximately 15% of people 60 years and older in the world living with a mental disorder, including anxiety and depression [12]. Given the well-established connections between social interactions and mental health in the elderly [13-16], the safety measures imposed due to COVID-19 may have had a negative impact on the mental health of this group. The mental health of the elderly is very important as it is closely related to their quality of life [17].

In the earlier phases of the emergence of COVID-19, China was the first to impose lockdowns and restrictions on movement and social gathering [18,19]. Chengmai County is a region in the touristic province of Hainan in China [20], famously known as the “Hometown of Longevity” since the life expectancy of its residents is 80 years, and 215 centenarians live in the region [21]. There are, however, 2 different communities of older people in Chengmai County that have different dialects, living habits, and socioeconomic characteristics: the local and the foreign community groups. The foreign group is composed of the so-called migratory birds, which is a colloquial term used to describe people, most often retirees, who reside in other regions of China but spend extended periods in Chengmai County during the winter months. Once COVID-19 restrictions were put into place, many migratory birds were stranded in Hainan. Although the epidemic in China subsided after the implementation of strict containment measures and movement restrictions [22,23], the effects of the COVID-19 pandemic on the mental health of older people from both communities in Chengmai County have not yet been investigated.

Although there are several studies demonstrating the high burden of anxiety and depressive symptoms among older people during the COVID-19 pandemic worldwide [24-30], the trend of anxiety and depressive symptoms has not been investigated in the region of China with the highest life expectancy. Therefore, the aim of this cross-sectional study was to investigate the effects of the COVID-19 pandemic on mental health and its contributing factors among older people from 2 different communities in Chengmai County, China.

## Methods

### Ethical Considerations

This work was undertaken by the Xinhua Hospital Affiliated to Shanghai Jiao Tong University School of Medicine, which approved the secondary use of the data for this publication (protocol number XHEC-D-2021-165). Informed consent was obtained from all subjects involved in the study. Participation in the survey constituted voluntary consent to participate. Responses were confidential.

### Study Design

This was a cross-sectional web-based study. Data collection was conducted from March 30, 2020, to April 9, 2020, via the web-based survey platform Wenjuanxing. The survey was disseminated through WeChat to local groups of hospital employees, schools, government organizations, and elderly individuals. All survey questions were in Chinese. Participants were asked about their circumstances in the previous month. Researchers were available to answer questions via WeChat or phone. Only 1 survey could be completed per device, and the link was active for 1 month.

### Sample

The sample was comprised of 2 communities of older people (ie, 50 years or older) in Chengmai County, China: a local and a foreign group. The local group was composed of people who were born in the region and have lived there since. The foreign group was composed of migratory birds (ie, people who reside in other regions of China but spend extended periods in Chengmai County during the winter months) or people who retired in this region. The exclusion criteria were the following: (1) people with severe cognitive impairments or any conditions that preclude them from being able to understand and agree to participate in the research; (2) people 50 years or older who are not retired; (3) people with severe liver and renal disease, malignant tumors, severe anemia, autoimmune diseases, or a history of acute cerebrovascular accidents; and (4) people unwilling to cooperate with the research protocol.

### Measures

The web-based survey consisted of the following measures: (1) participants’ clinical and sociodemographic characteristics, namely age, gender, marital status, living situation, educational attainment, fixed financial income, preretirement occupation, internet access, history of previous psychological disorders, and recent psychological trauma; (2) the presence of anxiety disorders measured by the 7-item Generalized Anxiety Disorder scale (GAD-7) [31]; and (3) the presence of depressive disorders measured by the 9-item Patient Health Questionnaire (PHQ-9) [32].

The GAD-7 is a 7-item instrument that measures symptoms of anxiety. Each item is scored on a 4-point Likert scale ranging from 0 (“not at all”) to 3 (“nearly every day”). Scores range from 0 to 27, with higher scores reflecting greater anxiety symptoms. Cutoff points of 5, 10, and 15 might be interpreted as representing mild, moderate, and severe levels of anxiety, respectively [31,33]. The GAD-7 has shown good reliability and validity in Chinese populations [34,35].

The PHQ-9 is a 9-item instrument that measures depressive symptoms corresponding to the diagnostic criteria for major depressive disorders. For each item, patients are asked to assess how much they were bothered by the symptoms over the previous 2 weeks. Each item is also scored on a 4-point Likert scale ranging from 0 (“not at all”) to 3 (“nearly every day”). Scores range from 0 to 27, with higher scores reflecting greater depression severity. Similar to the GAD-7, cutoff points are scores of 5, 10, and 15, which represent mild, moderate, and severe levels of depression, respectively [32]. The PHQ-9 has been considered valid and reliable to identify depression in the Chinese population [36].

### Statistical Analysis

Data were exported from Wenjuanxing to SPSS (version 22; IBM Corp), where the statistical analysis was performed. Descriptive statistics were used to describe participants’ socioeconomic and clinical characteristics. Chi-square tests were used to compare the proportions of respondents across the 2 community groups. The normality of the data distribution was tested using the Kolmogorov-Smirnov test. Independent *t* tests were used to investigate differences between anxiety and depressive symptoms across the 2 community groups. To identify the sociodemographic and clinical factors associated with anxiety and depressive symptoms in respondents from the 2 community groups, a multiple linear regression analysis was carried out with GAD-7 and PHQ-9 total scores as dependent variables and the collected demographic and clinical characteristics as independent variables. *P*<.05 was considered statistically significant for all tests.

## Results

### Respondents

Overall, 469 responses were received, of which 245 (52.2%) were from the local community group and 224 (47.8%) from the foreign community group. The overall sample was predominately elderly (321/469, 68.4% between 55 and 74 years), female (n=350, 74.6%), and married (n=409, 87.2%); most participants had high educational attainment (n=328, 69.9% of the sample had at least a high school degree). In addition, only 9.6% (45/469) of participants lived alone. Table 1 presents the sociodemographic and clinical characteristics of respondents overall and by community group. As described, respondents from the foreign community group were significantly older (*P*<.001), had higher educational attainment (*P*<.001), were more likely to have had a physical labor occupation before retirement (*P*<.001), had more fixed income (*P*<.001), relied more on their own expenses and less on medical allowances to pay for medical costs (*P*<.001), were more likely to live with their spouses (*P*<.001), used social media more often, and were more likely to have a history of mental health disorders (*P*<.001) and psychological trauma (*P*=.03) than respondents from the local community group.

**Table 1:** Sociodemographic and clinical characteristics for the overall sample and by community group.

Characteristic				Overall sample (N=469), n (%)		Local community group (n=245), n (%)		Foreign community group (n=224), n (%)		Chi-square value (*df*)		*P* value
**Age group in years**												
	50-54 years old			96 (20.5)		82 (33.5)		14 (6.3)		77.031 (4)		<.001
	55-64 years old			171 (36.5)		63 (25.7)		108 (48.2)				
	65-74 years old			150 (32)		63 (25.7)		87 (38.8)				
	75-84 years old			46 (9.8)		32 (13.1)		14 (6.3)				
	≥85 years old			6 (1.3)		5 (2)		1 (0.4)				
**Sex**												
	Male		119 (25.4)		57 (23.3)		62 (27.7)		1.204 (1)		.27	
	Female		350 (74.6)		188 (76.7)		162 (72.3)					
Married				409 (87.2)		209 (85.3)		200 (89.3)		1.661 (1)		.20
High school or higher education				328 (69.9)		127 (51.8)		201 (89.7)		79.922 (1)		<.001
Physical labor occupation before retirement				150 (32)		115 (46.9)		35 (15.6)		52.744 (1)		<.001
Fixed monthly income				341(72.7)		121 (49.4)		220 (98.2)		140.583 (1)		<.001
**Medical insurance**												
	Employee medical insurance			229 (48.8)		105 (42.9)		124 (55.4)		137.787 (2)		<.001
	Medical allowances			160 (34.1)		135 (55.1)		25 (11.2)				
	At one's own expense			80 (17.1)		5 (2)		75 (33.5)				
**Living situation**												
	Alone			45 (9.6)		11 (4.5)		34 (15.2)		211.12 (2)		<.001
	With spouse			233 (49.7)		57 (23.3)		176 (78.6)				
	With children			191 (40.7)		177 (72.2)		14 (6.3)				
History of mental health disorders^a^				28 (6)		5 (2)		23 (10.3)		14.108 (1)		<.001
Recent history of psychological trauma^b^				78 (16.6)		32 (13.1)		46 (20.5)		4.715 (1)		.03
Internet access				339 (72.3)		126 (51.4)		213 (95.1)		111.33 (1)		<.001

^a^Symptoms of anxiety or depression or a diagnosis of these conditions was considered a history of mental health disorders for this study.

^b^Psychological trauma that occurred in the past 3 months was considered recent.

### Presence of Anxiety and Depressive Disorders

Table 2 displays the presence of anxiety (measured by the GAD-7) and depressive disorders (measured by the PHQ-9). Overall, 20.5% (96/469) of respondents presented some levels of anxiety and 19.2% (n=90) presented depression. Regarding the differences between community groups, the mean GAD-7 (*P*=.003) scores were significantly higher in the local group compared to the foreign one. Anxiety was significantly more present in the local group compared to the foreign group (61/245, 24.9% versus 35/224, 15.6%; *P*=.01). There was no significant difference in the presence of depressive disorders between the groups. Finally, 6 respondents presented scores related to severe anxiety and 2 respondents presented scores related to severe depression.

**Table 2:** The presence of anxiety and depressive disorders in the overall sample and each community group.

Mental health disorder		Overall sample (N=469)	Local community group (n=245)	Foreign community group (n=224)	*P* value
**Anxiety**					
	GAD-7^a^ total scores, mean (SD)	2.3 (3.3)	2.7 (3.7)	1.8 (2.8)	.003
	Anxiety identified by GAD-7 scores (ie, >5), n (%)	96 (20.5)	61(24.9)	35 (15.6)	.01
	Mild anxiety (GAD-7 scores between 5 and 9), n (%)	79 (16.8)	49 (20)	30 (13.4)	—^b^
	Moderate anxiety (GAD-7 scores between 10 and 14), n (%)	11 (2.3)	7 (2.9)	4 (1.8)	—
	Severe anxiety (GAD-7 scores between 15 and 21), n (%)	6 (1.3)	5 (2)	1 (0.4)	—
**Depression**					
	PHQ-9^c^ total scores, mean (SD)	2.3 (3.2)	2.3 (3.3)	2.4 (3.0)	.73
	Depression identified by PHQ-9 scores (ie, >5), n (%)	90 (19.2)	50 (20.4)	40 (17.9)	.48
	Mild depression (PHQ-9 scores between 5 and 9), n (%)	70 (14.9)	39 (15.9)	31 (13.8)	—
	Moderate depression (PHQ-9 scores between 10 and 14), n (%)	18 (3.8)	9 (3.7)	9 (4)	—
	Severe depression (PHQ-9 scores between 15 and 27), n (%)	2 (0.4)	2 (0.8)	0 (0)	—

^a^GAD-7: 7-item Generalized Anxiety Disorder scale.

^b^—: Not available

^c^PHQ-9: 9-item Patient Health Questionnaire.

### Factors Associated With the Presence of Anxiety and Depressive Disorders

The regression models for the local and foreign community groups are presented in Multimedia Appendix 1 and Multimedia Appendix 2, respectively. Results suggest that a history of mental health disorders and psychological trauma are associated with anxiety (*F*_9,235_=2.45; *P*=.01; *R*^2^=0.09) and depression (*F*_9,235_=2.88; *P*=.003; *R*^2^=0.10) in the local community group. In the foreign community group, only having a physical labor occupation before retirement was associated with depressive disorders, although the overall regression model was marginally significant (*F*_9,214_=1.79; *P*=.07; *R*^2^=0.07).

## Discussion

This study was conducted to investigate the effects of the early months of the COVID-19 pandemic on mental health and its contributing factors among older people from 2 different communities in a Chinese region with one of the longest life expectancies. During the first months of the pandemic, in 2020, China was the first country that imposed strict restrictions, when little was known about the virus and its effect; therefore, results from this study describe the mental health effects in this scenario. The findings from our study identified that 20.5% (96/469) of respondents presented some levels of anxiety and 19.2% (90/469) presented depression, and anxiety was significantly more present in respondents from the local community group.

It is unanimously agreed that the COVID-19 pandemic has negatively impacted the mental health of different groups, such as health care providers [37-39], people living with disabilities or chronic conditions [5,40-43], pregnant women [44,45], young people [46], and the elderly [25,27,29,30,47]. Although with different characteristics, activities, routines, and lifestyles, the impacts of COVID-19 on these groups have led to an increase in loneliness, anxiety, depression, insomnia, alcohol and drug use, and self-harm or suicidal behavior [48]. Specifically in the elderly group, there has been an identified increase in loneliness in response to COVID-19 measures [49], which can lead to poor mental and physical health [50], serious illness, and mortality [51,52]. Although we have not assessed loneliness in this study, since only 10% (45/469) of respondents lived alone, it is possible that social interactions with spouses and family members living in the same household helped compensate for the decrease in other social interactions due to COVID-19 restrictions.

Results from this study suggest that a history of mental health disorders and psychological trauma are associated with anxiety and depression in the local community group. Therefore, approaches to help mitigate the negative impact of the COVID-19 pandemic on older people with previous mental health disorders are warranted. Interventions should focus on multiple components, including emotional, spiritual, social, and physical support to meet the various health needs of the elderly [53-55]. Internet-based interventions have demonstrated the potential to support the self-management of mental health conditions and could provide people access to information and tools during COVID-19 restrictions [56-58].

Caution is warranted in interpreting the findings of this study. First, the cross-sectional nature of this study allows no causal interpretation of the results. We are not able to identify if mental health disorders are further aggravated due to COVID-19 restrictions or if the results reflect preexisting differences in mental health. Moreover, we do not know what the delayed effects of COVID-19 in the following months and years are, and if these disorders were aggravated since restrictions continue to take place 2 years after the start of the pandemic. This should be evaluated in a future study. Second, the generalizability of the results is unknown and may be limited for the following reasons. We do not know how many people received the invitation to complete the web-based survey. In addition, this was a convenience sample, so the results may be biased. Third, although we have used well-established and validated tools to identify anxiety and depressive disorders, the gold standard to establish a clinical diagnosis of a mental disorder is a diagnostic structured interview with a professional. Finally, developing and applying appropriate interventions for mental health during a pandemic is essential. For this reason, future research in this area should focus on the design of appropriate interventions targeting improvements in mental health and the adoption of a healthy lifestyle during COVID-19 restrictions and beyond.

In conclusion, the present study investigated the mental health of older people living in the so-called Hometown of Longevity in China. Results showed that 20% (n/N) of respondents had anxiety or depressive disorders, and there was an association between current mental health status and a history of psychological disorders, highlighting the need to take measures to prevent, identify, and treat mental health problems in this group. Although many studies have been conducted focusing on mental health and COVID-19, the global crisis we are now living in is still new and rapidly evolving. The long-term consequences of this pandemic on mental health should be evaluated and effective mental health interventions should be developed.

## Appendix

Multimedia Appendix 1 Multiple regression analysis for socioeconomic and clinical factors affecting anxiety and depressive disorders in the local community group.

Multimedia Appendix 2 Multiple regression analysis for socioeconomic and clinical factors affecting anxiety and depressive disorders in the foreign community group.

## Abbreviations

GAD-7: 7-item Generalized Anxiety Disorder scale
PHQ-9: 9-item Patient Health Questionnaire

## Data Availability

Data are available on request due to privacy and ethical restrictions.
