# Supplementary material for: The Effects of the COVID-19 Pandemic on Mental Health Among Older Adults From Different Communities in Chengmai County, China: Cross-sectional Study
Source: JMIR Form Res. 2022 May 6;6(5):e37046. doi: 10.2196/37046 (PMC9084446; doi:10.2196/37046)
Supplement: Multimedia Appendix 1 [file formative_v6i5e37046_app1.docx]

Multiple regression analysis for socioeconomic and clinical factors affecting anxiety and depression disorders in the local community group

|  | **Anxiety** | | | | | **Depression** | | | | | |
| --- | --- | --- | --- | --- | --- | --- | --- | --- | --- | --- | --- |
| **Variables** | **β** | **SE** | ***P*** | **95% CI Lower Bound** | **95% CI Upper Bound** | | **β** | **SE** | ***P*** | **95% CI Lower Bound** | **95% CI Upper Bound** |
| Sex | .08 | .57 | .22 | -.42 | 1.82 | | .06 | .51 | .32 | -.49 | 1.51 |
| Age | -.08 | .27 | .36 | -.78 | .28 | | -.04 | .24 | .62 | -.60 | .36 |
| Education level | -.10 | .20 | .25 | -.63 | .16 | | -.03 | .18 | .72 | -.42 | .29 |
| Physical labor occupation before retirement | -.04 | .66 | .66 | -1.58 | 1.00 | | .10 | .58 | .24 | -.46 | 1.84 |
| Married | .01 | .70 | .91 | -1.31 | 1.46 | | -.05 | .63 | .45 | -1.71 | .76 |
| Fixed income | .05 | .57 | .50 | -.74 | 1.50 | | .07 | .51 | .38 | -.56 | 1.44 |
| History of mental health disorders ^a^ | .15 | 1.65 | .02 | .69 | 7.20 | | .19 | 1.47 | .002 | 1.63 | 7.42 |
| Recent history of psychological trauma ^b^ | .19 | .71 | .004 | .70 | 3.51 | | .18 | .64 | .006 | .51 | 3.52 |
| Internet access | .04 | .59 | .62 | -.87 | 1.46 | | .09 | .53 | .24 | -.42 | 1.66 |

SE, standard error; CI, confidence interval.

^a^ Mental health disorders: anxiety or depression symptoms or diagnostic.

^b^ Psychological trauma occurred in the past 3 months.
