# Supplementary material for: The Effects of the COVID-19 Pandemic on Mental Health Among Older Adults From Different Communities in Chengmai County, China: Cross-sectional Study
Source: JMIR Form Res. 2022 May 6;6(5):e37046. doi: 10.2196/37046 (PMC9084446; doi:10.2196/37046)
Supplement: Multimedia Appendix 2 [file formative_v6i5e37046_app2.docx]

Multiple regression analysis for socioeconomic and clinical factors affecting anxiety and depression disorders in the foreign community group

|  | **Anxiety** | | | | | **Depression** | | | | | |
| --- | --- | --- | --- | --- | --- | --- | --- | --- | --- | --- | --- |
| **Variables** | **β** | **SE** | ***P*** | **95% CI Lower Bound** | **95% CI Upper Bound** | | **β** | **SE** | ***P*** | **95% CI Lower Bound** | **95% CI Upper Bound** |
| Sex | -.03 | .45 | .69 | -1.07 | .71 | | -.06 | .47 | .39 | -1.33 | .52 |
| Age | .02 | .28 | .83 | -0.49 | .61 | | -.09 | .29 | .18 | -.95 | .18 |
| Education level | .02 | .24 | .38 | -0.27 | .69 | | .03 | .25 | .73 | -.41 | .58 |
| Physical labor occupation before retirement | -.04 | .63 | .61 | -1.56 | .92 | | -.16 | .65 | .04 | -2.63 | -.07 |
| Married | -.03 | .65 | .67 | -1.55 | 1.00 | | -.01 | .67 | .90 | -1.40 | 1.24 |
| Fixed income | -.02 | 1.51 | .77 | -3.42 | 2.54 | | .09 | 1.57 | .22 | -1.16 | 5.00 |
| History of mental health disorders ^a^ | .10 | .65 | .17 | -.38 | 2.16 | | .08 | .67 | .27 | -.55 | 2.08 |
| Recent history of psychological trauma ^b^ | -.08 | .48 | .23 | -1.52 | .37 | | .04 | .50 | .25 | -.66 | 1.30 |
| Internet access | .06 | .91 | .37 | -.97 | 2.62 | | .08 | .94 | .53 | -.81 | 2.90 |

SE, standard error; CI, confidence interval.

^a^ Mental health disorders: anxiety or depression symptoms or diagnostic.

^b^ Psychological trauma occurred in the past 3 months.
